# 

**Published:** 1898-04-30

**Authors:** 


					THE HOSPITAL. "The standard of highest purity."?Lancet. April 30th, 1898.
CADJouo?RY's ft H r? CADcBoVo5YS
DRUGS OR ALKALIES USED.
fC( ? S ^ ~
No- fins. Vnl YYTT7 rReffiatered aal o
No. 605. Vol. XXIY.
Saturday,
April 30th, 1898.
[8?b.?p??nT?.-j pBICE TW0pENCE

				

